# Every Wheeze Does Not Merit a Puffer! Case of an Overnight Cure of Chronic Asthma

**DOI:** 10.1155/2010/498372

**Published:** 2010-07-04

**Authors:** Santosh Balakrishnan, Sudeendra Doddi, Sophie Helme, Prakash Sinha

**Affiliations:** Department of General Surgery, Princess Royal University Hospital, Farnborough Common, Orpington, Greater London, BR6 8ND, UK

## Abstract

The peril of incorrect diagnostic labelling is highlighted by this case of acute respiratory distress caused by a retrosternal recurrent goitre. An initial clinical diagnosis which cannot be fully validated on investigation with unexpected or poor response to treatment should prompt consideration and investigation for an alternative explanation.

## 1. Introduction

Clinical diagnosis should be the summation of clinical history and examination followed by investigations to confirm the diagnosis and determine the aetiology. Once a diagnostic “label” has been given to an individual, one should be aware of the peril of continuing to accept this “label,” and not questioning the diagnosis. This is particularly important in a common disease such as asthma when a presentation is unusual and/or the response to treatment is not along expected lines.

## 2. Case Report

A 54-year-old man was referred by his GP to the accident & emergency (A&E) with acute onset stridor. At presentation he had a distressing audible biphasic (inspiratory and expiratory) stridor. He had no dysphagia. There were no obvious abnormalities noted on clinical examination of the upper airways and the lung. He was moderately hypoxic with O_2_ saturation of 92% on air on pulse oximetry. This improved to 98% with an increase of Fi02 to 25% by mask. 

He had been diagnosed with asthma eight years ago as a result of recurrent episodes of shortness of breath. Treatment with steroid (Beclometasone dipropionate) and ß_2_ agonist (Salbutamol) inhalers had provided him little relief. Peak flow measurements were recorded at 500 to 550 L/min which was in the normal range for his age and height. He was referred to a respiratory physician and a pulmonary function test was normal. He was returned back to the care of his GP. However, he continued to be treated with inhalers. He was also on treatment for epilepsy and had uneventful surgery seventeen years ago for a multinodular goitre and had been clinically euthyroid since. 

**Figure 1:**
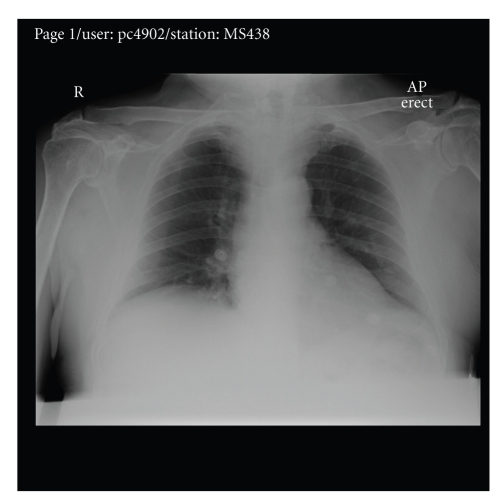
Chest X-Ray showing widened superior mediastinum.

**Figure 2:**
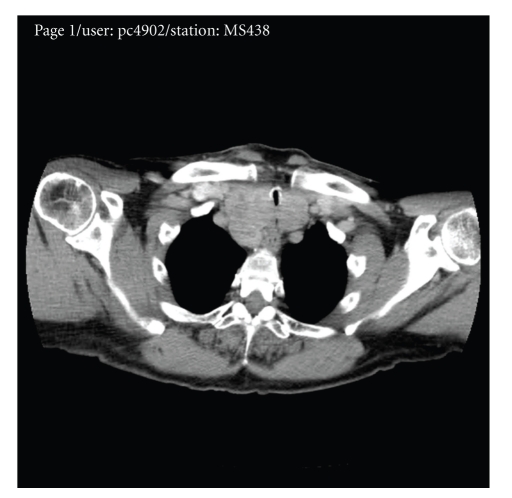
CT scan showing the retrosternal goitre compressing the trachea.

A chest X-ray (Figure 1) showed normal lungs (i.e., not hyperinflation) with a soft tissue shadow in the upper mediastinum. A CT scan showed a large right retrosternal goitre causing tracheal compression to a diameter of 3.7 mm (Figure 2). He underwent an emergency right thyroid lobectomy. The patient felt he had a miraculous cure overnight for his asthma of many years. Histology confirmed a benign multinodular goitre. 

Two years since his operation the patient continues to be euthyroid and symptom-free on a small suppressive dose of thyroxine aimed at preventing enlargement of the residual tissue from the left lobe.

## 3. Discussion

Asthma is a common condition and was considered as the first diagnosis. A late onset of asthma, a past history of thyroid problems, poor response to asthma medication, normal peak flow measurement, and lung function tests not suggestive of asthma should have prompted a search for an alternative diagnosis [1]. Wheezing may be caused by other diseases [2]. In a study of 153 consecutive patients with thyroid enlargement, 20% of the patients had upper airway obstruction clinically and on flow volume loop [3]. It would be prudent to limit dissection to the compressing lobe in an emergency situation in the presence of scarring from previous surgery and thereby minimise morbidity. 

## 4. Conclusion

This case highlights the need to rethink and not just accept the diagnosis if disease continues, the patient is not responding appropriately to usual clinical management and/or there are unusual features to the presentation.
